# Enhancing Transition to Workplace

**DOI:** 10.17795/nmsjournal17554

**Published:** 2014-04-17

**Authors:** Reza Negarandeh

**Affiliations:** 1 Nursing and Midwifery Care Research Center, Tehran University of Medical Sciences, Tehran, IR Iran

**Keywords:** Emotional Hardness, Workload, Nursing Education

Researches have demonstrated that new graduates encounter a degree of ‘‘emotional hardness’’ and cynicism during clinical placements, which can influence the quality of their provided care (1). Meleis (2010) defined transition as “a passage from one fairly stable state to another fairly stable one, which is a process triggered by a change” (2). Accordingly, experiencing transition seems to be inevitable for graduated nurses starting to work as a professional nurse and when they do and try to socialize effectively, they may feel uncomfortable and inadequate due to perceived lack of knowledge, skills, and support. Transition is a confusing and overwhelming incident causing stress, anxiety, fear and uncertainty (3). During transitions, nurses frequently encounter role conflict, marginalization and burnout. Inability to cope with transition may lead to job dissatisfaction, intention to leave their job and also may interfere with quality of care they provide (1, 4). Therefore, it is essential to recognize factors inhibiting successful transition. Pre-registration education is a major issue in the transition period from student to staff nurse. Despite the purpose of nursing educational systems to prepare nurses to provide the quality nursing care, in most countries including Iran, it seems that graduates of nursing programs were not well prepared for working independently (3). Similarly, employers and nursing managers believe the present educational systems are not able to train skillful nurses. It has been shown that approaches to preparing nursing students to make a successful transition into the workplace have not been effective enough. Developing dependent work behaviors rather than problem-solving skills in students by most faculties have affected students’ self-concept and self-confidence negatively, and decreased their self-esteem (5). At present, the baccalaureate program is the basic nursing program at the academic level and the only way leading to registration as a professional nurse in Iran (6). Our nursing education system has imposed several changes in nursing curriculum in the recent years like inclusion of internships to respond such criticisms. In addition, some nursing schools have implemented preceptorship and other programs as pilot projects in undergraduate nursing curriculum. The realities of nursing work environment are the other aspects strongly associated with successful transition from student to a professional nurse. Iranian nurses suffer from poor work conditions, high workload, low salary, limited clinical autonomy, conflicts with physicians, managerial issues and a lack of powerful supportive working conditions. Such poor working conditions are associated with burnout and high rates of turnover among nurses in Iran found to be related with both nursing education and clinical placements (7). On the other hand, nursing system in Iran faces many problems including severe shortage of manpower, and financial constraints. These two factors disturb programs which are invented to facilitate transitions to the new working environment such as orientation tours. In such circumstances, nurses have to work independently from their first working day without participating in preparation programs, while conducting orientation tours and providing written and verbal information are considered as important parts of the transition program. The importance of support for successful transition from student to a professional nurse is clear, but lack of effective supports is still of importance. Graduates short and long-term performance and therefore making decisions about new graduates remaining in the workforce could be seriously influenced by lack of professional and collegial support during the transition phase (4). As explained, factors such as lack of knowledge, skills, confidence and support can influence nurses’ ability to cope with transition. Therefore, continuing education (CE) programs could be useful to facilitate coping with transition. Unfortunately, Iranian nurses still allocate little time to CE due to pressing workload and shortage of personnel (6). The consequences of not paying attention to effective coping with transition become clear noticing that a significant proportion of nursing graduates do not work in nursing profession after few years, and continue to work or educate in fields other than nursing (4). This, along with the need of the country to more than 20 thousand new nurses has led to increasing admission in nursing schools as well as establishment of new education centers in the recent years. These programs may cause more difficulties in transition of graduated nurses to professional ones by reducing the quality of nursing pre-registration courses. Nursing managers and policy makers must adopt holistic approaches and refocus on efforts on all factors that hinder effective coping with transition to the working environment.
